# Review of Chemistry for Students, Adapted to the Courses as Taught in the Principal Medical Schools of the United States

**Published:** 1851-04

**Authors:** 


*Review of Chemistry for Students, adapted to the Courses as taught in the Principal Medical Schools of the United States.* By JNO. G. MURPHY, M. D. PHILADELPHIA: LINDSAY & BLAKISTON, 1851.

THIS is a neat little compendium of chemistry, which may be very useful to the student, in enabling him to keep up with lectures, and fix in his mind the teachings of the Professor. Chemistry has become an extensive and laborious study, and such books as this by Dr. Murphy are absolutely necessary for the medical student, who can devote comparatively little time to this particular branch of medical inquiry. Dentists who may wish to acquire a proper knowledge of chemisty, and others of them who may desire to revise their knowledge of this interesting science, will find a proper text book in this work.

